# Getting schooled: teachers’ views on school-based breastfeeding education in Lebanon

**DOI:** 10.1186/s13006-019-0199-8

**Published:** 2019-01-08

**Authors:** Sara Moukarzel, Christoforos Mamas, Antoine Farhat, Alan J. Daly

**Affiliations:** 1 Larsson-Rosenquist Foundation Mother-Milk-Infant Center of Research Excellence, University of California San Diego, La Jolla, CA 92093 USA; 2 Department of Education Studies, University of California San Diego, La Jolla, CA 92093 USA; 3 Faculty of Nursing and Health Sciences, Notre Dame University, Zouk Mosbeh, Lebanon

**Keywords:** Breastfeeding, Education, Schools, Teachers, Attitudes, Barriers, Lebanon

## Abstract

**Background:**

School-based breastfeeding education (SBBE) may help improve breastfeeding rates in the long term by instilling in young people a base of evidence-informed knowledge, skills, and attitudes that primes them to make informed decisions about infant feeding and to become positive change agents. Breastfeeding rates in Lebanon remain suboptimal, and breastfeeding misconceptions along with social pressures to use infant formula are known contributing barriers. We conducted this study with pre-K-12 teachers to understand the SBBE landscape as well as the supports and constraints for SBBE at two large Lebanese schools.

**Methods:**

We conducted a survey with 193 teachers during the 2017–2018 academic school year to collect information about demographics, breastfeeding history, breastfeeding teaching practices, attitudes towards SBBE such as attitude towards educating both boys and girls about breastfeeding, and views on potential constraints to successful SBBE implementation. Descriptive statistics and thematic analysis were used. Binary multiple logistic regression was used to ascertain the effects of teacher characteristics on likelihood to support SBBE.

**Results:**

While limited SBBE is currently taking place with only eight (4%) teachers reporting teaching about breastfeeding, 133 (69%) reported students should learn about breastfeeding in school. A multiple regression model [χ2(4) = 19.71, *p* = 0.001] showed teachers were more likely to support SBBE if they/their partners had ever breastfed, if they taught biology, or if they believed that schools should educate both boys and girls about breastfeeding in a society where discussing breastfeeding in public is a taboo. One hundred and ten (60%) teachers reported several concerns to SBBE implementation which included limited uptake by students who might not find SBBE valuable to them and resistance from parents due to cultural barriers. In order to effectively expand SBBE in these schools, 71 SBBE supporters (59.2% of respondents; 13 with missing data) suggested supporting local teachers to deliver SBBE, and 48 (40%) suggested mandating SBBE.

**Conclusions:**

Teachers held generally positive views on SBBE, which provides a fertile ground for growing SBBE in their schools. Future steps need to include engaging parents, students, and school principals to further understand the social constrains to SBBE before program design.

**Electronic supplementary material:**

The online version of this article (10.1186/s13006-019-0199-8) contains supplementary material, which is available to authorized users.

## Background

The importance of breastfeeding for maternal and infant health is well documented and has been widely disseminated in guidelines by the World Health Organization and others [1–4]. Breastfeeding promotion, support, and protection have been identified as instrumental for the achievement of the WHO Global Strategy for Women’s, Children’s and Adolescent Health (2016–2030) by helping to end all preventable maternal, infant, and child deaths and by ensuring the health and wellbeing of everyone before the year 2030 [5]. Exclusive breastfeeding is recommended for the first 6 months of life, with continued breastfeeding until at least 2 years of age [4]. Yet, exclusive breastfeeding rates remain low across many countries. In light of the need to increase exclusive breastfeeding rates globally [1, 2], recent efforts show promise when using an evidence-based multidimensional framework to assess countries’ readiness to and progress with scaling up and sustaining national breastfeeding promotion and support programs, particularly in low and middle income countries [6–8]. This important “top-down” approach to scaling up breastfeeding rates within a country largely involves developing policy recommendations for high-level decision makers who would then leverage, enable, or facilitate policy change.

Another complementary “bottom-up” approach to change involves efforts that address the underlying supports and constraints of breastfeeding within smaller groups of people at the community level. These include assessing and addressing individual-level barriers to breastfeeding such as knowledge gaps, negative beliefs and misconceptions among parents and their social ecosystem (i.e, family members, neighbors, friends, physicians) [9–18]. One of several long term strategies advocated to increase breastfeeding initiation, exclusivity, and duration is school-based breastfeeding education (SBBE) [4, 19–21]. The overarching goal behind SBBE, tailored towards improving breastfeeding knowledge and attitudes among children and adolescents, is to prime the future generation of parents to make informed decisions related to infant feeding [20, 22, 23]. Better informed students hold the potential to normalize breastfeeding not only by breastfeeding or supporting their partners to breastfeed in the future, but also by becoming agents of change in their communities, even if they do not become parents themselves [23–25]. While there is growing interest to expand the empirical base around SBBE, little attention has been given to the breadth and depth of SBBE, along with its supports and constraints, in schools within developing nations which are often underserved, under-supported, and under-researched.

Given the importance of the topic and potential of change, we examined the breastfeeding education landscape in a developing nation (Lebanon), where around 38% of infants are exclusively breastfed during the first month of life and around 2% are exclusively breastfed at 6 months of age [26, 27]. Individual-level barriers to breastfeeding include lack of sufficient knowledge about why and how to breastfeed as well as negative beliefs and misconceptions among mothers, their family members, and physicians [17, 18, 27–29]. Examples of these misconceptions include the notions that women are biologically incapable of breastfeeding if their mothers were not able to successfully breastfeed them, that women must not be providing sufficient amounts of human milk if their infants are crying, and that abdominal cramps can be transferred from mother to infant through human milk [17, 18, 27–29]. Additionally, breastfeeding remains controversial in the Lebanese society as a whole, as it is often perceived as a female-only issue. Most mothers who breastfeed struggle to overcome social barriers to breastfeed in public and to continue breastfeeding after returning to work [17, 18, 30]. While women are entitled to 10 weeks of paid maternity leave, many anecdotally choose to return to work earlier and to stop breastfeeding in fear of losing promotions and even employment. Indeed, the absence of comprehensive legislation and policies in Lebanon against sexual exploitation and harassment continue to contribute to discrimination against women in the workplace [31]. Accordingly, there is a clear large social component that contributes to suboptimal breastfeeding rates in Lebanon and possibly to suboptimal maternal and infant health.

The role of education is widely recognized as an instrument of social change and social development, beyond mere knowledge transmission [32–34]. School-based health education programs show promise for the prevention or promotion of several issues (i.e, substance abuse and chronic diseases, promotion of physical activity and a healthy dietary lifestyle), one of which is breastfeeding [23, 35–38]. This is in part because school-based education instills in the youth a base of knowledge, skills, beliefs, and values that can extend into adulthood [35]. Within the breastfeeding space, several studies outside of Lebanon have reported that students at various ages have negative beliefs about breastfeeding and insufficient knowledge about infant feeding recommendations, as well as the maternal and infant benefits of breastfeeding [23, 24]. Breastfeeding-related beliefs among Lebanese students are not currently known, but negative beliefs may exist as students are likely be influenced by their larger social ecosystem.

Short term SBBE interventions have generally resulted in increased knowledge, improved beliefs, and increased intention to breastfeed in the future [23, 25]. Therefore, SBBE may be one instrument to help normalize breastfeeding in the long term in Lebanon. There is currently no documentation related to if, what, how, and when students are learning about breastfeeding in Lebanese schools. Effective SBBE in Lebanon requires understanding of the supports and constraints of SBBE curriculum design and implementation, which are largely unknown and possibly school-specific [39, 40]. Teachers are key stakeholders who hold the potential to fill these knowledge gaps and inform strategy development aimed at incorporating or improving SBBE in their schools. Educator knowledge, personal experiences, and beliefs related to breastfeeding and to SBBE may be important contributors to shaping the mindsets around breastfeeding of the future generation [24, 41, 42]. We therefore conducted this study with pre-K-12 teachers to better understand the SBBE landscape at two large Lebanese schools, as well as the supports and constraints for SBBE.

## Methods

### Setting

This study was conducted in two purposefully selected private schools in the governorate (province) of Mount Lebanon in Lebanon. Given the exploratory nature of this work, we selected schools that have economically diverse student populations, compared to public schools that mainly serve students with lower socioeconomic status. The majority of private schools in Lebanon serve students from low, middle, and high income families, unlike in many countries where private schools are only affordable to students from high income families. Accordingly, it is likely that students in private schools more closely represent the population compared to public schools that predominantly serve students with lower socioeconomic status. More than half of all schools in Lebanon are private (54%). The most recently available data shows 103,660 students were enrolled in Pre-K-12 schools in Mount Lebanon, of which 74% were enrolled in private schools [43]. Adult literacy rate in Lebanon is 90%. Around 97% of boys and girls are enrolled in primary schools, but more girls than boys are enrolled in secondary schools (80 and 77%, respectively) [44].

### Sample and data collection

This study was conducted during the 2017–2018 academic year. Participant recruitment took place at the schools with collaboration from division principals. One hundred and ninety-three of a total of 241 teachers completed the study and the response rate was 80%. Participants anonymously completed a survey, grounded in previous work [22], that captured information about demographic characteristics, breastfeeding history, current breastfeeding teaching practices, and attitudes towards breastfeeding and towards SBBE. Singletary et al. [22] developed a validated and reliable survey to assess each teachers’ current education practices and attitudes towards infant feeding education in schools, guided by a literature review [24] and by results from qualitative interviews with teachers [22]. The interviews and survey questions targeted individuals who specifically teach Family and Consumer Sciences classes in North Carolina in the United States, and in our study, we modified questions related to current breastfeeding education practices to capture practices across all school grades and by all teachers independent of which classes they teach. Questions used to describe the current SBBE landscape are summarized in Table 1. To identify SBBE supporters, we included the yes/no question “Should students learn about breastfeeding in school”. Additionally, to learn more about how SBBE supporters envisioned incorporating SBBE in their schools, we a list of all subjects taught across all grades (Table 3) to choose one or more subject in which breastfeeding should be taught. They were then asked to match the chosen subject (s) to the grade level (s) they find most fitting. Other questions were also used to assess SBBE supporters’ views on how to implement SBBE in their schools (Table 2). There is no previous work published within this research space in Lebanon and we used our best judgement, based on our personal experiences as educators and on conversations with Lebanese Pre-K12 educators to provide answer options that might be relevant to pre-K-12 education in Lebanon, adding the “other” option to capture unlisted information.

**Table 1:** List of questions used to identify current teaching practices among participants who reported teaching about breastfeeding and to identify reasons for not teaching about breastfeeding among others

1) In which grades are you teaching about breastfeeding? [half an empty line on A4 paper format was provided] 2) Circle the statement that best applies to you: ● It is mandatory to teach about breastfeeding in my class. ● It is not mandatory to teach about breastfeeding in my class. 3) What specific areas related to breastfeeding do you teach about in your class? Circle all that apply: ● Human milk composition ● Human milk benefits to the infant ● Human milk benefits to the mother ● For how long women are recommended to exclusively breastfeed ● Physiological barriers and contraindications to breastfeeding ● Psycho-social barriers to breastfeeding ● Other: [one empty line] 4) Circle the statement that best applies to you: ● I only teach about breastfeeding in my class without promoting breastfeeding (eg. I don’t encourage students to breastfeed/support breastfeeding when they have their own children) ● I teach about breastfeeding and promote breastfeeding in my class. 5) Circle the statement that best applies to you: ● I teach about breastfeeding using a lecturing format. ● I teach about breastfeeding using an active learning activity (e.g. project, game, class discussion) ● I teach about breastfeeding using a lecturing format and an active learning activity. 6) Circle all the reasons why you do not teach about breastfeeding in your class: ● I am not required to teach about breastfeeding. ● The topic of breastfeeding is not relevant to the subject I teach. ● I do not have time to spare in class to teach about breastfeeding. ● I do not know enough about the topic of breastfeeding. ● I do not know how to teach about breastfeeding. ● I do not feel comfortable teaching about breastfeeding. ● I do not have enough teaching material to use in class. ● Other: [one empty line]

**Table 2:** List of questions used to explore SBBE supporters’ views on how to implement SBBE in their schools

1) Who should be providing breastfeeding education in your school? ● Teachers alone ● Teacher with a health professional as invited guest to class (circle all that apply) ○ Dietitian ○ Nurse ○ Physician ○ Midwife ○ Other: [Half an empty line] ● Health professional alone (circle all that apply) ○ Dietitian ○ Nurse ○ Physician ○ Midwife ○ Other: [Half an empty line] ● Other: [One empty line] 2) Do you have a strict curriculum to follow or are you free to teach extracurricular topics in class? [two empty lines for additional details] 3) Which one of the following would be most effective in expanding breastfeeding education in your school? ● Convince and support teachers to include breastfeeding education in their classes. ● Adding breastfeeding as a required topic in the curriculum. ● Other: [one empty line]	

We also developed seven Likert-type self-report statements (six options from “strongly disagree” to “strongly agree”) to assess attitudes towards breastfeeding and SBBE, as we were unable to find published studies that identify the beliefs and attitudes of teachers in Lebanon specifically. The statements were inspired from previous work where SBBE was proposed as a means to help future parents make an informed decision about infant feeding practices and to promote positive attitudes towards breastfeeding [22, 24], as well as from studies predominately with pregnant and lactating women which report that positive beliefs about the benefits of breastfeeding to both maternal and infant health are important determinants of breastfeeding intention and behavior [45–47]. An Exploratory Factor Analysis (EFA) with principal component extraction and Varimax (orthogonal) rotation of items was conducted. The purpose of the EFA was not to validate a scale to use on another sample, but to assess the reliability of these items for the current study only. Two items did not load well on any component and were therefore eliminated. The items were: *Breastfeeding provides the infant with health benefits* and *Deciding that one’s infant be breastfed is a personal matter that should not be advocated in schools.* The resultant attitude scale factored into two components: The First component encompasses beliefs around the potential of SBBE to help normalize breastfeeding in Lebanon. It includes three items: *Breastfeeding provides health benefits to the mother*; *Educating students about breastfeeding will help them make an informed decision about breastfeeding their own children later on in life;* and *Educating students about breastfeeding will promote positive attitudes towards breastfeeding in the Lebanese society*. The second factor includes two items: *It is a taboo to discuss the topic of breastfeeding in public* and *Breastfeeding should not only be taught to girls in school.* The Kaiser-Meyer Olkin test of the sample indicated that the items could be factorable (KMO = 0.702), and the Bartlett’s Test of Sphericity was significant (*p* < 0.001). Cronbach’s alpha demonstrated fairly strong reliability across items (alpha = 0.758).

The resultant survey was available in paper form, in three languages (Arabic, French, and English), and an identical format across languages to optimize response rate as suggested by several teachers. Survey items were reviewed for face validity by members of the research team, which included three teacher-scholars, three academic researchers whose areas of focus include the study of social determinants of and barriers to breastfeeding, one school principal, and two high school biology and English language teachers. The survey was informally piloted before administration with a convenient sample of *n* = 21 teachers (*n* = 7/survey language) to make sure questions are easy to understand and complete.

### Statistical analysis

The distributions of continuous variables were tested using Kolmogorov-Smirnov test and were found to be normal. Descriptive statistics were used, and results are expressed as means ± standard deviations (SD), frequency and percentage, or median (range) as appropriate. Binary multiple logistic regression modelling was performed to predict the relationship between being supportive of SBBE (Answered “yes” to “should students learn about breastfeeding in school”) and teacher characteristics. SPSS software (version 25 for Mac OS X) was used, and the level of statistical significance was set at *p* < 0.05. Responses to the open-ended survey question pertaining to identifying challenges related to SBBE implementation were coded on MAXQA 2018 (VERBI Software, 2017) and analyzed through thematic analysis starting with open-coding [48]. All responses were reviewed independently by two researcher members who both identified the same four major challenges/themes from the overall responses. The researchers reviewed their descriptions of the challenges together and revised the descriptions to reach consensus before reporting.

## Results

### Current SBBE landscape (the what, when and How’s)

A total of 193 teachers (*n* = 91 from school A and *n* = 102 from school B) completed the study. Their descriptive characteristics are summarized in Table 3. Limited SBBE was reported in both schools, with minor differences between schools in their education curricula (See Additional file 1). In school A, it is mandatory to teach only about the infant and maternal benefits of breastfeeding and only in the 5th grade life sciences class. In school B, it is mandatory to teach about human milk composition, as well as breastfeeding benefits, recommendations and barriers, but only in 10th grade. Although not mandated to do so, three additional life sciences teachers and one teacher in social studies choose to teach about various aspects of breastfeeding. All eight participants (4%) who teach about breastfeeding reported also promoting breastfeeding in their classes.

**Table 3:** Descriptive characteristics of participating teachers

Characteristics, *n* (%)	Descriptive statistics
Demographic variables	
Gender	
Female	168 (87)
Male	25 (13)
Age, years^a^	40 ± 11
Children	
Has at least one child	148 (77)
No children	45 (23)
Number of children^b^	2 (0–5)
Age of eldest child, years^b^	12 (0.25–42)
Age of youngest child, years^b^	9 (0.5–35)
Gender of children	
Only has males	40 (27)
Only has females	33 (22)
Has children of both	75 (51)
Breastfeeding characteristics	
Breastfeeding history	
Ever breastfed^c^	116 (78)
Never breastfed	32 (22)
Feeding of youngest child	
Only human milk	33 (22)
Human milk and formula	84 (57)
Only formula	27 (18)
Missing data	4 (3)
Breastfeeding experience^d^	
Unpleasant	16 (14)
Neutral	13 (11)
Pleasant	81 (70)
Missing data	6 (5)
Teaching characteristics	
School division	
Pre-K and Kindergarten	38 (20)
Primary (1st- 3rd grade)	41 (21)
Elementary (4th- 6th grade)	31 (16)
Intermediate (7th – 9th grade)	37 (19)
Secondary (10th – 12th grade)	46 (24)
Teacher by subjects taught^e^	
Languages	106 (55)
Chemistry, physics, and/or math	51 (26)
Biology^f^	33 (17)
Social studies^g^	18 (9)

Participant characteristics and study results did not differ by school, results were pooled and presented for one cohort. ^a^mean ± SD; ^b^median (range); ^c^either participant or partner has breastfed any of their children, exclusively or not, for any duration of time during the first 6 months postnatal; ^d^*n* (%) corresponds to those who have ever breastfed or have a partner who ever breastfed; ^e^teacher may teach more than one subject i.e., many of biology teachers also teach languages and math; ^f^class is referred to as “life sciences” in 1st to 9th grade; ^g^includes sociology, economics, philosophy, civics, history, religion, geography, and art

### Constraints and supports for SBBE

#### Constraints for SBBE

Overall, the majority (*n* = 185) do not teach about breastfeeding, including *n* = 26 (78%) of biology and life sciences teachers. At least one of the following reasons were identified among biology and life sciences teachers: first, there is no requirement to do so (54% of teachers), second, some teachers do not find breastfeeding as a relevant topic to teach (46%), and third, a few do not have enough breastfeeding teaching materials (2%). Teachers for all other subjects reported not teaching about breastfeeding predominantly because of a perceived irrelevance of the breastfeeding topic to the subjects they teach (67%) and because there is no mandate to do so (26%). Other reasons included not having sufficient knowledge or enough teaching materials.

One hundred and ten teachers (60%) responded to the open-ended question “what challenges do you anticipate teachers might face related to teaching about breastfeeding in your school”, with two empty answer lines provided. While *n* = 37 wrote “none”, the remaining teachers identified four common potential challenges. First, teachers were concerned that knowledge uptake by students of all ages would be limited either because students will not find SBBE valuable to them (i.e, not interested in the topic) or because students might consider the topic too sensitive to discuss in class. For example, one 28-year old female teacher with no children noted, “Since it [breastfeeding] is somewhat of a taboo, students might feel embarrassed or shy or might laugh. Eventually they will adapt just like when they learn about the menstrual cycle.” Second, teachers mainly in the pre-K and primary divisions voiced concerns about the maturity level and readiness of their students to learn about breastfeeding at young age. Third, the potential opposition to SBBE by their fellow teachers was reported as a barrier. Reflective of this sub-group of teachers, one 47-year old female teacher who has three children and has reported having breastfed noted, “The [breastfeeding] topic is part of private life within a family. We can risk having the topic misinterpreted or misunderstood if we talk about it.” Finally, teachers were concerned about resistance from parents against SBBE due to the cultural barriers. For example, one 25-year old female teachers with no children wrote “parents, culture and [their] lack of open-mindedness”. Therefore, in addition to school-level structural constraints, the social and cultural environment within which SBBE is implemented may have important implications to whether SBBE will be applicable and effective.

#### Predictors of SBBE support

A large proportion of teachers (*n* = 133, 69%) reported that students should be learning about breastfeeding in school. These teachers referred to as SBBE supporters were more likely to have a personal breastfeeding history: 62% of SBBE supporters reported having breastfed compared to 56% of SBBE non-supporters (Table 4). Of note, percentages without each group of supporters and non-supporters do not add to a 100 for breastfeeding history because percentages are only calculated for teachers with children. Additionally, the following teachers were more likely to be SBBE supporters than non-supporters: those who teach in high school-level classes, who currently teach about breastfeeding, as well as teachers who believe that discussing the topic of breastfeeding in public is a taboo and that breastfeeding should be taught to both boys and girls in school (Table 4) .

**Table 4:** Teacher characteristics found to be significantly different between SBBE supporters and non-supporters

Variable, *n* (%)	SBBE supporters *(N* = 133)	SBBE non-supporters *(N* = 57)	*p* - value
Breastfeeding history			
Ever breastfed^a^	82 (62)	32 (56)	0.015
Never breastfed	14 (11)	17 (30)	
High school teacher			
Yes	38 (29)	8 (14)	0.013
No	95 (71)	49 (86)	
Currently teaching about BF			
Yes	8 (6)	0 (0)	0.044
No	125 (94)	57 (100)	
Attitude score^b^	5.39 ± 0.59	5.18 ± 0.68	0.048

SBBE, school-based breastfeeding education*.* BF, breastfeeding. *n* = 3 participants did not respond to whether or not they support SBBE (missing data)*. p -* values determined using Chi-square test except for attitude score determined using independent student t - test. ^a^either participant or partner has breastfed any of their children, exclusively or not, for any duration of time during the first 6 months postnatal; ^b^mean ± SD with a maximum possible score of 6 for strongly agreeing that the discussing the topic of breastfeeding in public is a taboo and that breastfeeding should be taught to both boys and girls in school

A binary multiple logistic regression was performed to ascertain the collective effects of teacher characteristics and attitude scores towards SBBE on the likelihood that a teacher thinks breastfeeding should be taught in school. The variables included in the model (Table 3) were largely based on previous findings in the literature [22, 24]. After removing the most statistically insignificant characteristics and re-coding two variables (classes taught [high school/ not high school classes], and subjects taught [Biology or life sciences/all others]), the logistic regression model presented in Table 5 was statistically significant, χ2(4) = 19.71, *p* = 0.001. The model explained 20.5% (Nagelkerke R^2^) of the variance and correctly classified 92% of cases. Despite odd ratios being small, the findings do show that after accounting for other variables in the model, teachers were more likely to support SBBE if they/their partners had ever breastfed, teach biology, or if they believed that schools should educate both boys and girls about breastfeeding in a society where discussing breastfeeding in public is a taboo.

**Table 5:** Binary multiple logistic regression analysis of the effect of teacher characteristics on likelihood of supporting SBBE

Variables	B	S.E	Wald	df	*p*	Odds Ratio	95% Confidence Interval	
Breastfeeding History [1 = yes, 2 = no]	−1.216	0.482	6.356	1	0.012	0.296	0.115	0.763
Mean score on attitude scale^a^	0.760	0.321	5.613	1	0.018	0.468	0.249	0.877
Biology teacher [1 = yes, 2 = no]	−1.340	0.638	4.412	1	0.036	0.262	0.075	0.914
Highschool teacher [1 = yes, 2 = no]	−0.957	0.539	3.147	1	0.076	0.384	0.134	1.106
Constant	4.539	1.762	6.639	1	0.010	93.643		

^a^The attitude scale is a continuous variable with a minimum of 1[strongly disagree] and maximum of 6 [strongly agree] assessing to what extent a teacher believes that it is a taboo to discuss the topic of breastfeeding in public in Lebanon and that schools should teach both boys and girls, not just girls, about breastfeeding

#### Windows of opportunity

We were interested in exploring how best to implement SBBE from the perspective of teachers who were supportive of SBBE in their schools (*n* = 133). The majority (*n* = 81, 61%) suggested SBBE be implemented for high school-level students as part of biology class for students to learn about human milk composition, maternal and infant benefits of breastfeeding, as well as the physiological barriers and contraindications to breastfeeding. In addition to a biology related focus areas, several teachers proposed that SBBE be included in sociology (reported by *n* = 15 SBBE supporters), civics (*n* = 5), and religion (*n* = 5) classes to also address the psychosocial and cultural breastfeeding barriers within the Lebanese society. Of note, while the majority stated SBBE is best implemented as part of high school classes*, n* = 43 (63%) teachers from the elementary and intermediate divisions stated that their students (4th to 9th graders) could benefit from learning about the biological and social aspects of breastfeeding as well.

Independent of class level and content, SBBE supporters predominantly suggested that SBBE be delivered not by the teacher alone, but also by a health professional as an invited guest to class (131 out of 133 SBBE supporters; 98.5%). In decreasing order of frequency, SBBE supporters suggested inviting a dietitian (47%), nurse (38%), physician (34%), or midwife (24%). Here, the sum of percentages is greater than 100 as SBBE supporters could provide more than one option for health professionals. Finally, although the general K-12 national education curricula across grades are mandated by the government, most SBBE supporters (*n* = 78, 67.8% of respondents; *n* = 18 missing data) reported having the freedom to teach extra curriculum topics in class. Of the 78 teachers, 40 (51.3%) wrote in the provided space following this question (Table 2) that they can teach extra curriculum topics if they can make time for that in class. Therefore, in order to effectively expand SBBE in these schools, *n* = 71 SBBE supporters (59.2% of respondents; *n* = 13 missing data) suggested convincing and supporting local teachers to deliver SBBE in their classes. Alternatively, *n* = 48 (40%) suggested adding breastfeeding as a required topic in the curriculum and one participant suggested doing both.

## Discussion

School culture, which comprises the school’s values and norms, is highly influenced by the school principal and teachers and is known to affect school’s areas of teaching priorities and students’ academic performance [49, 50]. Beyond academics, Pre-K-12 schools play a potentially important role in shaping students’ character, values, and ultimately behavior [34, 51, 52]. As such, SBBE programs have the potential to serve the promotion, support, and protection of breastfeeding through a variety of ways. These include disseminating evidence-informed knowledge to students about the implications of breastfeeding to maternal and infant health, economic aspects and social support. It might be equally necessary to support students in developing positive attitudes and skills to make informed decisions about infant feeding based on scientific evidence and to help normalize breastfeeding in their communities. For example, previous skill-based health education school programs have focused on improving attitudes toward responsibility for personal, family, and community health as well as improving students’ confidence to change habits [53]. Those same processes may be applied to breastfeeding education. Other skills can also include the ability to communicate evidence-based messages around breastfeeding to families, peers, and members of the community [53].

Despite the potential of education, we still need formative research to guide the design, implementation, and evaluation of a feasible and effective SBBE program that is tailored to the needs of the students (and community around them) and that capitalizes on or overcomes existing school cultures [40]. Similar to many Pre-K-12 educational curricula around the world [23], we found limited SBBE in the two large Lebanese schools, which serve over 7000 students. Our findings suggest that the mandatory curricula around breastfeeding may be limited and only include basic information about the nutritional composition of human milk and breastfeeding health benefits. Physiological and psychosocial barriers to breastfeeding are sometimes included as topics of discussion in class, but the teachers did not report educating their students that evidence-based solutions are available and accessible for many of the barriers. It would be interesting to further explore the extent to which solutions to breastfeeding barriers are taught using one-on-one interviews or focus groups. Additionally, there does not seem to be much focus around breastfeeding within the Lebanese societal context and how to possibly address these issues.

Our results suggest that SBBE may be a powerful catalyzer of change. More than two-thirds of the teachers were supportive of having SBBE implemented in their affiliated schools. This finding aligns with results from few previous studies we could find on teachers’ attitudes towards SBBE (*n* = 210 combined, from the US and Nigeria) [22, 54, 55]. Buy-in from Lebanese teachers to develop and implement an SBBE program likely stems from their general belief in the maternal and/or infant benefits of breastfeeding, and in the need for and the ability of school based education to help normalize breastfeeding [22, 24]. Interestingly, 25 teachers from all school divisions and various disciplines (i.e, languages, biology, chemistry, sociology) envisioned SBBE as a fitting theme in classes other than biology. Their vision to expand SBBE to sociology, religion, and civics studies stimulates our interest into exploring SBBE program design using problem-based learning and interdisciplinary teaching approaches.

Teachers most supportive of SBBE were mainly biology teachers and other teachers who have at least one child that was breastfed and who hold more fact based beliefs about breastfeeding. Of note and contrary to our speculations, we did not find any significant effects of gender or breastfeeding experience (unpleasant/neutral/pleasant) on the likelihood of supporting SBBE. However, the latter may be explained by the fact that study participants were predominantly females (~ 90%) and that the majority (70%) did report their/their partners’ breastfeeding experience was pleasant. The high proportion of females in our study is reflective of the well-known gender imbalance in the Pre-K-12 teaching profession in Lebanon [56], although the most recent report from 2012 for female teachers in the Lebanese public sector is lower (75%) than in our study [56]. We did not explore the effect of teacher religion on breastfeeding beliefs and views towards SBBE which needs to be addressed to enhance confidence in the generalizability of our findings. Al-Sahab et al. [57] reported maternal religion as a strong predictor of breastfeeding among *n* = 1320 Lebanese mothers, where Muslims were twice as likely to be breastfeeding at 4 months compared to Christians. We surveyed teachers from two schools in Mount Lebanon with predominantly Christian residents. There is no accurate census data on religion distribution in Lebanon, but it is estimated that 54% of the population is Muslim, 40.4% is Christian, and 5.6% is Druze [58]. Whether religion also influences teachers’ attitudes towards SBBE merits further investigation. Regardless, findings from this first phase of our exploratory research suggest that, in both schools, teachers held generally positive views on SBBE, which provides a potentially fertile ground for growing SBBE in the breastfeeding promotion, protection, and support space. Accordingly, future engagement of school leaders (eg. school principals) and capitalizing on their leadership roles will be particularly important if the short term path forward will be to work with local teachers directly on incorporating SBBE in their classes, instead of working with influencers on national education policy, as the majority of teachers had suggested.

Although generally supportive of SBBE, teachers raised several concerns and envisioned challenges for the successful implementation of SBBE in their schools. Perhaps unsurprisingly, the majority of these involved some aspect of social pressure, such as the potential resistance from parents or the students themselves because breastfeeding “is somewhat of a taboo” and “is part of private life within a family”, as two teachers noted. The maturity level of the students and their ability to address the topic seriously was also of concern and consistent with previous work [22]. Therefore, in addition to having assessed teachers’ views on SBBE, assessing and addressing parents’, students’, and principals’ views on breastfeeding and on SBBE may be a promising area of investigation. Indeed, strong partnerships between schools and the community (i.e, students’ parents and families), built on mutual understanding, trust, and active involvement in students’ education, are well-known to enhance academic achievement and student motivation to learn and succeed [59].

Our results should be interpreted while taking into account several limitations, including the fact that our survey was reviewed for only face validity. We did assess the reliability of our attitude scale, but this should be considered exploratory. The attitude scale was not designed for reproduction on other samples, but simply to understand what was being measured in our case. Future studies should continue to validate these items with larger samples. Furthermore, the use of open-ended survey questions, in addition to multiple-option multiple-choice questions, is not equivalent to conducting in-depth qualitative exploration of the study subject [60, 61]. One-on-one interviews and focus groups with teachers are likely to provide more robust data and may unravel additional supports and constraints to SBBE implementation.

## Conclusions

In summary, we found teachers across school divisions and disciplines at two Lebanese schools to be keen to implement SBBE not only as part of biology classes but also in social studies to start the conversation around the roles of breastfeeding beyond health. Therefore, next short term steps will include engaging teachers and other education stakeholders (parents, students, and school principals) from these schools to further understand the social constrains to SBBE before any program design. Finally, we chose our point of departure as two private schools where students are predominantly Lebanese (> 99%). They typically stay at the same school from Pre-K classes until completion of Grade 12 which provides us with the unique opportunity to follow, intervene, and/or study the short and long term impact of SBBE in almost the same cohort of students across Pre-K-12 education. Our long term plans include research in a wider sample of public Lebanese and other developing nations schools that mainly serve students from low-income families.

## Additional file

Additional file 1: Summary of when, what and how breastfeeding-related topics are being taught in two Lebanese schools. (DOCX 19 kb)

## Abbreviation

SBBE: school-based breastfeeding education
