# Supplementary material for: Getting schooled: teachers’ views on school-based breastfeeding education in Lebanon
Source: Int Breastfeed J. 2019 Jan 8;14:3. doi: 10.1186/s13006-019-0199-8 (PMC6325822; doi:10.1186/s13006-019-0199-8)
Supplement: Supplementary file 1 — Summary of when, what and how breastfeeding-related topics are being taught in two Lebanese schools. (DOCX 19 kb) [file 13006_2019_199_MOESM1_ESM.docx]

**Additional file 1**

**Summary of when, what and how breastfeeding-related topics are being taught in two Lebanese schools**

| **Class** | **Grade** | **Areas of focus** | **Approach** | **Teaching method** | **Mandatory to teach?** |
| --- | --- | --- | --- | --- | --- |
| **SCHOOL A** | | | | | |
| Social studies | 11 | - Human milk composition - Physiological barriers and contraindications to BF | Teach evidence and promote BF | Lecture | No |
| Life sciences | 8 | - Human milk composition - BF benefits to infant - Recommended duration for exclusive BF - Physiological barriers and contraindications to BF | Teach evidence only without promoting BF | Lecture | No |
| Life sciences | 6 | - BF benefits to infant | Teach evidence only without promoting BF | Lecture | No |
| Life sciences | 5 | - BF benefits to infant | Teach evidence and promote BF | Active learning | Yes |
| Life sciences | 5 | - BF benefits to infant | Teach evidence and promote BF | Active learning | Yes |
| Life sciences | 5 | - BF benefits to infant and mother | Teach evidence and promote BF | Active learning | Yes |
| **SCHOOL B** | | | | | |
| Biology | 10 | - Human milk composition - BF benefits to infant and mother - Recommended duration for exclusive BF - Physiological barriers and contraindications to BF - Psycho-social and cultural barriers | Teach evidence and promote BF | Lecture | Yes |
| Biology | 6 & 7 | - Human milk composition - BF benefits to infant - Psycho-social and cultural barriers | Teach evidence and promote BF | Lecture | No |

BF, breastfeeding.
